# The Physicians Prescription Book: Containing List of Terms, Phrases, Contractions and Abbreviations Used in Prescriptions, with Explanary Notes; Also the Grammatical Construction of Prescriptions, Etc., Etc.

**Published:** 1851-07

**Authors:** 


### Part 2—Reviews and Notices of New Works.

ARTICLE I.

*The Physicians Prescription Book:* containing List of Terms, Phrases, Contractions and Abbreviations used in Prescriptions, with Explanary notes; also the Grammatical Construction of Prescriptions, etc., etc. To which is added a Key, containing the Prescriptions in an Unabreviated form, with a Literal Translation, intended for the use of Medical and Pharmaceutical Students. First American from the tenth London edition. Philadelphia: Lindsay & Blakiston: 1851: pp. 288: 12 mo: (from the publishers, through S. C. Griggs & Co., Chicago.)

This is an exceedingly convenient little work, filled with valuable information to the practitioner. And, as it is becoming more and more fashionable to dispense with the study of Latin in the preliminary education of students, the translations of Latin prescriptions, placed in parallel colums with them, will be very convenient to those, who wish to inform themselves of their meaning and construction, without the study of the language.

The definitions given of the Nomenclature used in prescriptions, will be useful to the medical student in acquiring a knowledge of medical technology.

Not less important, is the chapter on abbreviations. There are so many errors in the abbreviations of prescriptions, that attentton should be directed to them with a view to the means of theii prevention. Unquestionably this chapter will prove profitable to both the prescribing physician and the apothecary.

The formulary for powders, pills, etc., may be well for examples, but we cannot conceive that much advantage can arise from the study of a long catalogue of prescriptions given entirely independent of their therapeutical application.

Altogether, we regard the little work before us as calculated to be highly useful to the student, the physician and apothecary.

